# Clonidine to the Rescue: A Novel Approach to Refractory Diabetic Gastroparesis in the Postoperative Setting

**DOI:** 10.7759/cureus.79769

**Published:** 2025-02-27

**Authors:** Marsida Kasa, Merita Rroji, Nereida Spahia, Grisilda Gjana, Brunilda Elezi

**Affiliations:** 1 Department of Internal Medicine, University Hospital of Trauma, Tirana, ALB; 2 Department of Nephrology, University of Medicine, Tirana, ALB; 3 Faculty of Medical Technical Sciences, University Aleksander Xhuvani, Elbasan, ALB

**Keywords:** acute renal injury, chronic diarrea, diabetic complications, fluid status, hypertension and therapy

## Abstract

Gastroparesis is a recognized complication in patients with long-standing diabetes mellitus (DM), characterized by delayed gastric emptying without mechanical obstruction. Common symptoms include nausea, vomiting, bloating, and early satiety, all of which can significantly impair both quality of life and glycemic control. Standard therapies, such as prokinetic agents (e.g., metoclopramide and domperidone) and antiemetics (e.g., ondansetron), are commonly used but may fail in refractory cases, prompting an investigation into alternative treatments. Clonidine, an α2-adrenergic agonist traditionally prescribed for diabetic diarrhea, has demonstrated promise in managing autonomic dysfunction and thus may represent a novel option for gastroparesis management.

This case report describes a 65-year-old man with type 2 DM and stage 3 chronic kidney disease (CKD), presumed secondary to diabetic nephropathy, retinopathy, and peripheral neuropathy. Following orthopedic surgery, he developed severe postoperative vomiting, leading to the discontinuation of his antihypertensive therapy. After two days without antihypertensive medications, a hypertensive crisis occurred (blood pressure, 210/120 mmHg), accompanied by an acute rise in serum creatinine to 5 mg/dL, oliguria, and generalized edema. The ongoing vomiting further contributed to dehydration and worsening acute renal dysfunction. Despite receiving continuous veno-venous hemodialysis (CVVHD) for 48 hours and antiemetic treatment with metoclopramide and ondansetron for one week, his nausea and vomiting persisted. Other etiologies were excluded, and a diagnosis of diabetic gastroparesis was established. Initiation of clonidine at a dose of 100 mcg twice daily produced marked symptom improvement within 48 hours, enabling the resumption of regular oral intake and stabilization of renal function. The patient was discharged in stable condition and remained asymptomatic at his six-month follow-up appointment.

This case underscores the potential of clonidine as an adjunct treatment for patients with refractory diabetic gastroparesis, highlighting its impact on autonomic regulation and symptom alleviation. Further studies are warranted to substantiate its efficacy and safety in larger patient populations.

## Introduction

Gastroparesis is a significant gastrointestinal disorder in individuals with chronic diabetes mellitus (DM). Its prevalence is estimated at 5.2% in type 1 DM and 1% in type 2 DM over a decade, notably higher than the 0.2% observed in the general population [1,2]. Current evidence suggests that obesity, especially among individuals with neuropathy, increases the likelihood of gastroparesis in type 2 DM [3]. Additional risk factors include elevated HbA1c levels, a disease duration exceeding 10 years, and vascular complications [4].

The multifactorial pathophysiology encompasses autonomic neuropathy, smooth muscle dysfunction, and disturbances within the enteric nervous system and hormonal pathways [5]. Autonomic neuropathy disrupts the balance of sympathetic and parasympathetic input to the gastrointestinal tract, leading to motility abnormalities. Persistent hyperglycemia, vascular changes, and obesity further aggravate the condition [4]. Glucagon-like peptide-1 (GLP-1) has recently drawn attention, as GLP-1 agonists (e.g., exenatide, liraglutide) have been linked to the onset or worsening of gastroparesis [4].

Clinically, patients frequently report nausea, vomiting, bloating, and early satiety, which can compromise nutrition and complicate glycemic control. However, some studies question the direct association between delayed gastric emptying and these symptoms, indicating a more complex etiology [6]. Standard care typically involves dietary modifications and medications such as prokinetic agents (e.g., metoclopramide, domperidone) and antiemetics. Yet, these measures often fail in refractory cases, highlighting the need for innovative treatments.

Recent findings indicate that clonidine, an α2-adrenergic agonist commonly used for diabetic diarrhea, may offer a viable adjunct therapy for gastroparesis [5]. The following case report illustrates its use in a postoperative patient who developed severe, refractory diabetic gastroparesis complicated by acute kidney injury (AKI).

## Case presentation

Here, we present the case of a 65-year-old man with a 20-year history of type 2 diabetes mellitus, presumed diabetic nephropathy (chronic kidney disease (CKD) stage 3b, baseline serum creatinine 1.7 mg/dL, estimated glomerular filtration rate (eGFR) 44 mL/min/1.73 m²), diabetic retinopathy, and neuropathy who underwent open reduction and internal fixation of a left bimalleolar ankle fracture. One year prior to the current admission, he required hospitalization for three weeks due to severe vomiting precipitated by a viral infection, for which he received ondansetron. Six hours postoperatively, the patient experienced severe vomiting that precluded adherence to his antihypertensive regimen. After two days, he developed hypertensive urgency (blood pressure, 210/120 mmHg) and acute kidney injury (AKI) superimposed on CKD. Notably, he was not receiving any opioid analgesics; his only analgesic was acetaminophen. The nausea and vomiting began before any significant elevation in serum urea and persisted at the same intensity on the following days. On postoperative day three, his serum creatinine increased to 5 mg/dL, accompanied by elevated central venous pressure (CVP, 14 mmHg), oligoanuria, edema, dyspnea, hypoxemia, and ongoing gastrointestinal symptoms. While the patient was receiving metoclopramide and ondansetron, continuous veno-venous hemodialysis (CVVHD) was initiated and maintained for 48 hours, during which six liters of ultrafiltrate were removed. This intervention improved diuresis, corrected fluid balance, and partially restored renal function (with serum creatinine stabilizing at 4 mg/dL and urea at 150 mg/dL five days after discontinuing CVVHD). However, refractory nausea and vomiting persisted despite prokinetic and antiemetic therapy, compromising oral intake of both nutrition and antihypertensive medications and ultimately contributing to suboptimal blood pressure control. The main follow-up laboratory parameters are presented in Table 1.

**Table 1:** The main follow-up laboratory parameters of the patient CRP: C-reactive protein.

Parameter	Day 3 Post-Surgery	Day 10 (5 Days Post-CRRT)	At Discharge	Reference Range/Normal Values
Serum creatinine	5.0 mg/dL	4 mg/dL	2 mg/dL	0.6–1.2 mg/dL
Urea	240 mg/dL	150 mg/dL	110 mg/dL	07–20 mg/dL
Potassium	5.5 mEq/L	4.1 mEq/L	3.2 mEq/L	3.5–5.0 mEq/L
Sodium	134	141 mEq/L	139 mEq/L	135–145 mEq/L
pH	7.29	7.35	7.35	7.35–7.45
Bicarbonate	17 mEq/L	24 mEq/L	25 mEq/L	22–28 mEq/L
Lactate	2.5mmol/L	1.7 mmol/L	1.1 mmol/L	<2mmol/L
Hemoglobin	10 g/dL	10.5 g/dL	11 g/dL	12.1–15.9g/dL
CRP immuno	0.59 mg/dL	1.1 mg/dL	0.8 mg/dL	0–0.5mg/dL
Glycemia	165mg/dL	150 mg/dL	141 mg/dL	74–100mg/dL
Diuresis	0.1 ml/kg/h	1.8 mL/kg/h	2 mL/kg/h	>0.5 mL/kg/h
Central venous pressure	14 mmHg	9 mmHg	7 mmHg	>0.5 mL/kg/h
Blood pressure	210/120 mmHg	165/90 mmHg	120/80 mmHg	<120/80 mmHg

The failure of metoclopramide and ondansetron for a week prompted further investigation. Upper endoscopy ruled out mechanical obstruction, but bedside ultrasound was inconclusive due to the patient's inability to ingest test meals.

Following a thorough evaluation of potential etiologies, including uremic gastroenteropathy, postoperative ileus or mechanical obstruction, medication-induced vomiting, infectious or metabolic causes, and other upper gastrointestinal disorders, diabetic gastroparesis was ultimately diagnosed. Clonidine, traditionally used for diabetic diarrhea, was prescribed at 100 mcg twice daily. Within two days, nausea subsided, oral intake of food and medications was reestablished, urine output increased to five liters per day, creatinine decreased to 2 mg/dL, and blood pressure was reduced to 120/80 mmHg. The patient was discharged in stable condition, with clonidine integrated into his long-term regimen. At a six-month follow-up, he reported complete resolution of nausea and vomiting, stable blood pressure, and stable renal function.

## Discussion

This case emphasizes clonidine's efficacy in addressing refractory diabetic gastroparesis, enabling clinical recovery in a patient unresponsive to conventional therapies.

Gastroparesis is relatively common yet often underrecognized in long-standing DM. It is characterized by delayed gastric emptying without mechanical obstruction, posing diagnostic and therapeutic challenges, especially when compounded by other end-organ complications. From a clinical standpoint, suspicion should arise in patients presenting with erratic glycemic control, recurrent hypoglycemia, or alternating episodes of hyperglycemia and hypoglycemia, which are conditions driven by poor coordination between insulin action and carbohydrate absorption [5].

Approximately one-third of gastroparesis cases exhibit persistent symptoms with intermittent exacerbations, whereas another third follows a path of progressively worsening manifestations [4]. Early identification and effective management are critical to reducing complications. Autonomic dysfunction has been identified as a key contributor to the multifaceted pathophysiology [4].

Standard therapy typically involves prokinetic agents (e.g., metoclopramide), antiemetics (e.g., ondansetron), and dietary modifications. However, these strategies may be insufficient in severe or refractory presentations. Metoclopramide can alleviate nausea and vomiting but may not normalize gastric emptying, implying that persistent vomiting in diabetic patients is not solely attributable to motility defects [7]. Another agent, relamorelin, a ghrelin receptor agonist, has shown promise in improving gastric motility and lessening symptoms such as nausea and vomiting, emerging as a possible therapeutic resource for diabetic gastroparesis [8].

Clonidine, an α2-adrenergic agonist commonly used for hypertension and diabetic diarrhea, has more recently been explored for diabetic gastroparesis [9]. Its benefits derive from modulating sympathetic and parasympathetic pathways, potentially enhancing gastric motility and symptom relief. In the present case, clonidine markedly reduced nausea, enabling regular food intake and representing a novel therapeutic option for gastroparesis management.

Although clonidine lacks overt prokinetic actions at standard doses, research suggests a single oral dose of 0.3 mg may mitigate nausea and vomiting by targeting sensory or central neural pathways [10]. Thus, its therapeutic reach may extend beyond limited effects on motility and instead address sensory and autonomic components implicated in diabetic gastroparesis.

Implications and future research

In this case, clonidine's efficacy is a potential adjuvant therapy for diabetic gastroparesis, particularly in cases refractory to standard treatments. Additional investigations are required to define its specific role in enhancing gastric motility, mechanisms of action, and comprehensive generalizability in subjects with diabetic autonomic neuropathy. Mechanistic studies and a promising clinical trial will be necessary to establish its utility, determine dosing, and increase the armamentarium available to treat this complex disease.

## Conclusions

This case represents a novel use of clonidine for the treatment of diabetic gastroparesis in a patient with CKD and postoperative complications. Clonidine's effectiveness in alleviating refractory nausea and improving overall clinical stability highlights its potential as an alternative treatment and underscores the need for further investigation into its role and efficacy in the management of gastroparesis.
